# Spinouts bring women in engineering together — here’s why

**DOI:** 10.1038/s44172-024-00228-x

**Published:** 2024-06-24

**Authors:** Brittany Harris, Jade Cohen, Irena Tyshyna, Anna Baldycheva

**Affiliations:** 1 Quasiflow, London, UK; 2 CODA:GENE, London, UK; 3 Engineering Department, University of Exeter, Exeter, UK

## Abstract

This International Women in Engineering Day (INWED 2024) *Communications Engineering* celebrates women in innovative startups.

It is a known challenge to be a female researcher in engineering. It requires even more courage to translate your research to the business environment. In such circumstances, it is not surprising that women often choose to partner up when launching a startup, in order to share the challenges and victories in equal measure along the way. Today, we want to provide a perspective from the female co-founders of two spinout companies and explore their journeys to building a successful business. We will meet Brittany and Jade, whose company aims to improve sustainability in construction, and Anna and Irena, who bring Emotional AI to enhance digital learning environments.

**Figure:**
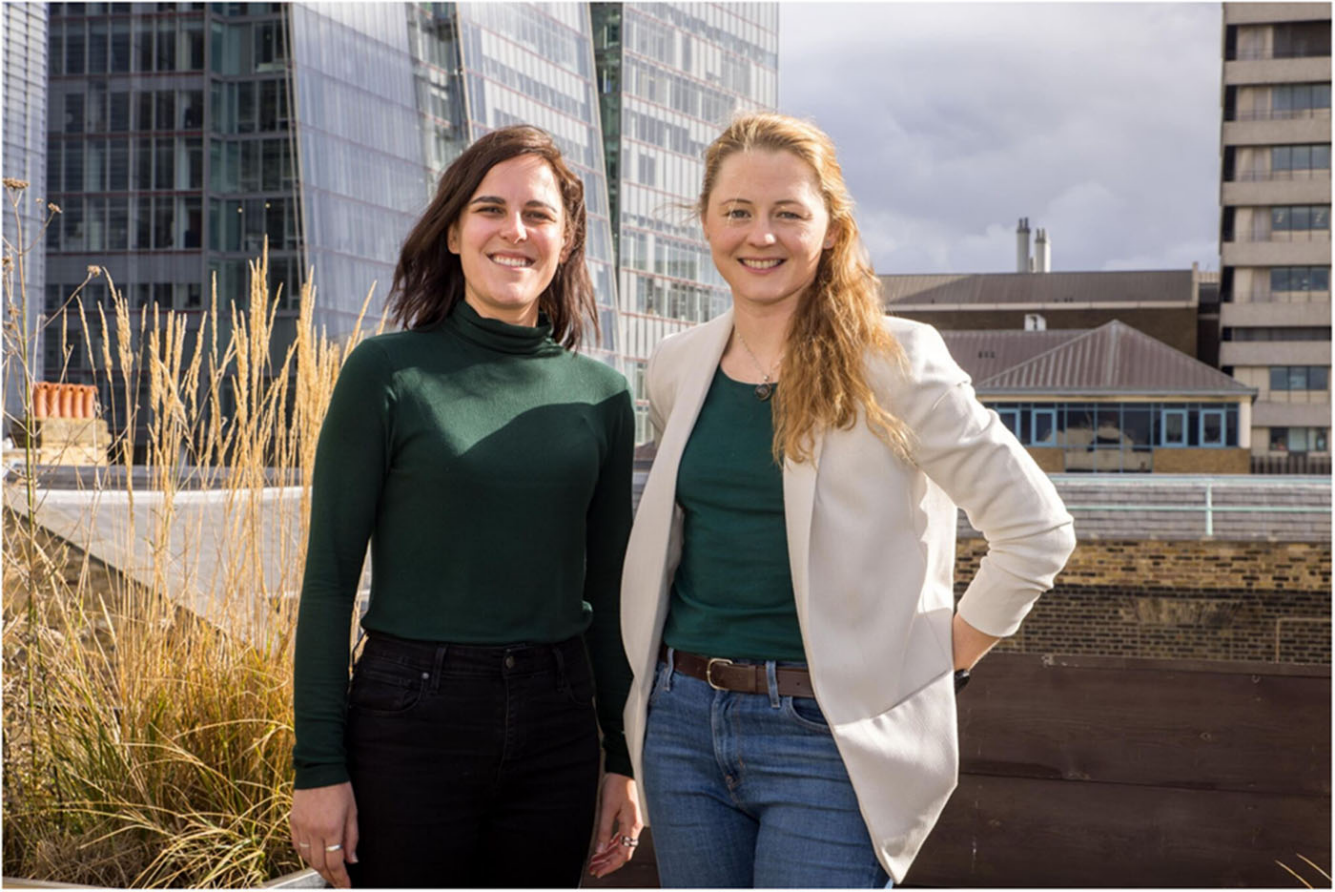
Meet Brittany Harris, Co-founder and CEO (right), and Jade Cohen, Co-founder and CPO (left). Qualis Flow.

**Tell us about your company’s first steps. What was your motivation to start and carry on with the challenge of launching a startup?**

**B.H. and J.C.:** We founded Qflow in 2018, with the ambition of transforming this linear, wasteful and pollutive industry into one that is circular and regenerative. We have grown from a team of 2 passionate young women, using our skills and connections in construction to tackle ethically sourced timber management, to a 51 strong team of software engineers, data scientists, and commercial and operational professionals, delivering a tool that helps construction teams track all possible materials, from concrete and steel, to light fittings and carpet tiles. Qflow has serviced over 200 construction sites across the UK, Australia and now the USA. This work has helped avoid over 9,690 tonnes of carbon emissions, avoided 31,000 tonnes of waste, and saved project teams over 5000 h of work.

We first presented the idea of Qflow to the UK’s Royal Academy of Engineering in 2017. Back then, we were still in full time jobs, and this was a fun side project. As the idea gained traction, we had to think long and hard about whether we would leave the security of a career in construction and engineering and go full-time on Qflow. We took the leap in March 2018, joining Entrepreneur First programme, which cushioned the leap with a stipend for 3 months while we further developed the idea.

**Describe your academic career: what research topics did you study and at what institutions?**

**B.H.:** I pursued my studies at the University of Bristol, UK, where I completed a Master of Engineering in Civil Engineering from 2011 to 2015. I focused on Water Engineering, Geotechnics and International Development with my 4th year thesis on alternative sanitation solutions for rural, low-income communities.

**J.C.:** I studied for a Master’s degree in Environmental Science at the University of Southampton, UK, which gave me a good foundation in understanding environmental law and core sustainability principles. I focused during my Master’s on looking at urban microclimate modelling, and how we can better design public realm spaces for adapting the urban and natural environment. However, outside of my studies I worked to establish social enterprises focused on tackling clean water and sanitation issues in other parts of the world. It was through these experiences that I gained my first taste of what it takes to start-up a new concept and bring this to life.

**Tell us how you met and decided to launch a spinout together**.

**J.C.:** Brittany and I met in New York, whilst volunteering for an organisation called World Merit. This involved a 2-week intensive program working with young professionals from across 21 countries to identify challenges and solutions to delivering the UN Sustainable Development Goal 6 – Clean water and Sanitation.

We bonded over our mutual love of all things water and sustainability, and quickly found mutual ground in construction. We continued to work on our spin-out not-for-profit project ‘Washable International’ for 2 years but found that we could not scale it in the necessary way. When Brittany came across the Launchpad competition run by the Royal Academy of Engineering, it seemed like a great opportunity for us to explore a ‘profit with a purpose’ model. Knowing that we had complimentary skills and personalities and worked well together, Brittany reached out to see if I would join her in this adventure.

We were both working in the AEC (architecture, engineering, construction) industry at the time; Brittany as a Civil Engineer for an engineering consultancy, and me as an Environmental Advisor for a construction company. We had an equal love-hate relationship with this industry; seeing both how impressive feats of engineering could shape our world, whilst being one of the most carbon intensive and wasteful sectors at the same time. We thought that we could change this, but only by affecting it from the ‘outside in’. We saw specific problems with the way that data was collected on construction sites, which inhibited efforts to minimise waste and work more efficiently. We therefore decided to tackle it problem-first, with the idea of how to solve this being refined over many months after that point.

**Tell us a story about external support during your spinout launch, how you were seeking support and how it helped you**.

**J.C.:** When we first started Qflow, I think it is fair to say that we did not quite know what we were getting ourselves into! Whilst we would both gain a fair amount of experience in the AEC industry and our independent sustainability pursuits, we were keen to seek support from others who had built a quick scaling, venture capital-backed business.

We sought out mentors and other founders we knew were going on this journey, and this community was enormously helpful as we started out and remains so today. We also now give back to this community – through independent mentorship to early-stage founders, or to other founder forums and networks. A particular focus of mine is to work with young, female founders, who can typically lack the confidence to take the deep dive into startup life or are struggling with their own leadership styles.

**Do gender-related considerations impact your experience of being a founder? It could be the way you lead the team, or interaction with customers or partners, or simply a part of your service**.

**B.H.:** The immediate answer is yes. Externally, we will often have male colleagues asked if they are the founders. We had to explain to investors that we are not planning to go off and have a baby. We also must work extra hard to convince people of the numbers and robustness of our plans, and even after that, we can get told that we are not visionary enough.

Internally, we aim to lead with compassion and put the team first. This can sometimes be taken advantage of, and is a hard line to walk, but we strongly believe this builds a better more sustainable culture and a great work environment. We take a very human and people first approach, as opposed to a tech-first approach.

**J.C.:** One of the hardest things to identify internally, is what our leadership is, and what we want it to be. The companies and leaders who are typically more acclaimed in the media, are male founders, with very male-dominant traits (think of characters historically like Steve Jobs or Elon Musk). Our leadership style is very different, and we exhibit more compassion, care, and patience with our team and the way we work, which we believe is fundamental to building a successful business, but are typically seen as female-orientated traits, and not as ‘strong’ as male-orientated traits when it comes to society’s traditional sense of what leadership is.

We do not believe this traditional style is right or necessary. However, this approach can be met with some surprise and scepticism, particularly if someone has worked in a company where the culture has not been as open and collaborative as the one we have successfully built with Qflow.

**What advice would you give to someone who is considering to follow the same path of starting a spinout?**

**B.H.:** My strong advice would be to always know why you are doing what you are doing. Your drivers need to be beyond financial returns, there is not a lot of financial security in the early years, and there may never be. You will need a strong ‘why’ to get you through the tough times. It may sound cheesy, but for us, this ‘why’ is the belief that we can have a more sustainable construction industry in the future. It is the belief that we can achieve a circular economy at scale, knowing that it takes mad, unreasonable people to commit their time and energy to deliver that. This is the reason why Jade and I get up every day, even when it can be hard, to push through and achieve our goals.

**J.C.:** I can only echo Britt’s comments in saying it’s all about the ‘why’. Why are you working on this particular idea? Why do you believe in it? Why is it worth taking this risk?

Alongside having this core mission at heart, I believe the key is also finding the right fit in a co-founder. It is not just about having complementary skill sets, it is also about sharing the emotional load, because it will never get any lighter over time! Me and Britt are opposites in many ways, and we use this to our advantage; we balance each other and get the best out of each other. This puts us in the best position for long-term success.

**Figure:**
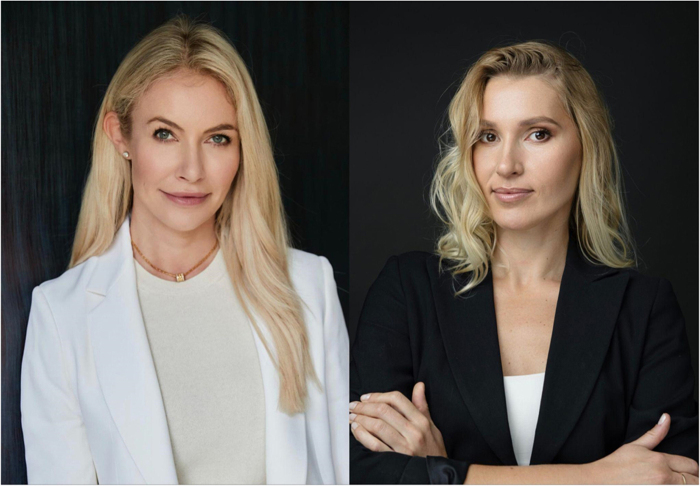
Meet Irena Tyshyna, Director and Co-founder (left), and Anna Baldycheva, Co-founder (right). Irena Tyshyna and Anna Baldycheva.

**Tell us about your company’s first steps. What was your motivation to start and carry on with the challenge of launching a startup?**

**I.T and A.B.:** Our project, launched in June 2023, aims to integrate Artificial Emotional Intelligence (Emotional AI) into digital learning environments. Our goals are to provide accessible, personalised education, build competitive skills, and equip educational institutions with efficient tools for digital delivery. By addressing limitations in online learning, such as reduced interpersonal communication and real-time feedback, we aim to enhance the digital learning experience through Emotional AI. in this project, we involve expertise from engineering, psychology, AI, and education teams to create a more engaging and effective learning process.

**Describe your (academic) career: what research topics did you study and at what institutions?**

**I.T.:** I am an educator and coach with over 25 years of practical experience and academic research in mental genetics, inherited behavioural patterns, mind hygiene, and cognitive reprogramming. I got a Master’s degree in Education in 2001 in Odesa, Ukraine. I then specialised in mental genetics, geno-physics, child psychology, and childhood studies. In 2018, I founded my consultancy practice, CODA:GENE, blending extensive practical experience with broad academic research to create a unique and effective educational method. My work in this project focuses on personalised online education through AI-assisted emotional analysis. My research involves collaborations with leading institutions to optimise learning experiences based on individual behavioural patterns. Balancing a career and single parenthood has enriched my understanding of flexible and inclusive learning environments, which I integrate into my consultancy and research projects.

**A.B.:** I completed my BSc (Hons) in Physics at Saint Petersburg University in 2008 and my PhD in Electronic and Electrical Engineering at Trinity College Dublin in 2012. My postdoctoral research at the Massachusetts Institute of Technology focused on electronics and microsystems technology. In 2014, I took a faculty position at the University of Exeter and founded the STEMM Laboratory - a highly interdisciplinary academic research lab working on applied research and development of smart materials, devices and systems, which produced several innovations and a technology spin-out. I also lead various initiatives to support women in STEMM and serve as an academic editor and a trustee in several scientific communities.

**Tell us how you met and decided to launch a spinout together**.

**A.B.:** We met at the Artificial Emotional Intelligence seminar in 2022, organised in my STEMM Laboratory. Our shared passion for integrating AI with emotional intelligence in education sparked numerous conversations and collaborative ideas. Recognising our complementary expertise — mine in engineering and AI, and Irena’s in psychology and education — we saw the potential to create a transformative project.

This project required a multidisciplinary approach, blending technological innovation with deep psychological insights to address the emotional and social aspects of digital learning. Our collaboration was born out of a mutual respect for each other’s fields and a shared vision of enhancing online education through Emotional AI. We officially joined forces in 2023 when Irena joined the STEMM Lab, and brought her qualifications and extensive experience in emotional intelligence and education to the table. Our decision to co-found the project was a natural progression of our successful collaborative efforts and our aligned goals.

**Tell us how you build mutual support to carry on through the company launch journey**.

**A.B.:** Despite the short period since the launch of our project, we quickly learned the importance of open communication and mutual support. Early on, we faced significant hurdles, numerous logistical and technical challenges. We developed a habit of regular check-ins and honest discussions, improving problem-solving and deepening our trust.

My technical expertise and Irena’s psychological insights complemented each other, enabling us to tackle issues from multiple perspectives. Celebrating small victories maintained morale, while empathy and flexibility with schedules fostered a resilient partnership. By staying in close communication and being transparent about our challenges and goals, we built a strong foundation of mutual support, ensuring our ongoing collaboration and growth.

**Do gender-related considerations impact your experience of being a founder? It could be the way you lead the team, or interaction with customers or partners**.

**I.T.:** Gender-related considerations do influence our experience as co-founders, particularly in a male-dominated field like engineering. However, these challenges have also shaped our leadership style to be more intuitive and empathetic. We prioritise creating an inclusive environment that values diverse perspectives, which we believe enhances our innovative capabilities. Our experiences have taught us the importance of resilience and adaptability, and we strive to lead by example. By fostering a supportive and inclusive culture, we aim to set a standard in the tech industry that demonstrates the strengths of diverse leadership.

**What advice would you give to someone who’s considering to follow the same path of starting a spinout?**

**I.T.:** The first thing would be to prioritise clear and honest communication with your co-founders and team. Establishing trust and mutual support is essential for overcoming obstacles. Seek out mentors and advisors who can provide guidance and perspective. Stay committed to your vision and be prepared to adapt your strategies as needed.

Let your core vision guide you, but adapt your approach to meet new challenges and opportunities. Being open to evolving your methods while keeping the ultimate goal in sight is crucial. Additionally, master organisational skills, professional responsibilities, and time management. Efficiently balancing these aspects is essential for maintaining progress and focus. This is a key for sustaining and growing your venture.

**A.B.:** I would also mention to stay curious and open to interdisciplinary collaboration. The most innovative solutions often come from the intersection of different fields. Building a diverse team with complementary skills is crucial for tackling complex problems. You may look at challenges as learning opportunities and maintain a clear focus on your goals. Persistence and adaptability are key to navigating the uncertainties of a startup journey.

